# Decision-making Factors in Surgical Techniques and Attitudes Towards Environmental Sustainability

**DOI:** 10.1097/SLA.0000000000006691

**Published:** 2025-03-11

**Authors:** Kim E. van Nieuwenhuizen, Herman J. Friedericy, Anne C. van der Eijk, Frank Willem Jansen, M.E. van den Akker-van Marle

**Affiliations:** * Department of Obstetrics and Gynaecology, Leiden University Medical Centre, Leiden, The Netherlands; † Department of Anaesthesiology, Leiden University Medical Centre, Leiden, The Netherlands; ‡ Operating Room Department, Leiden University Medical Centre, Leiden, The Netherlands; § Central Sterile Supply Department, Leiden University Medical Centre, Leiden, The Netherlands; ¶ Department of Biomedical Engineering, Technical University Delft, Delft, The Netherlands; ‖ Department of Biomedical Data Sciences, section Medical Decision Making, Leiden University Medical Centre, Leiden, The Netherlands

**Keywords:** surgery, carbon footprint, decision-making, sustainability, discrete choice experiment

## Abstract

**Objective:**

Our study examines factors influencing surgical specialists’ choice of surgical technique and assesses the significance of the carbon footprint in this decision-making process. It also investigates their attitudes, behaviors, and barriers to environmental sustainability.

**Background:**

Climate change significantly threatens health, with surgery being a major contributor to health care’s carbon footprint.

**Methods:**

A cross-sectional study was conducted using a discrete choice experiment (DCE) and a questionnaire. Respondents were Dutch-speaking surgeons, gynecologists, and urologists with experience in minimally invasive surgery. They judged 14 choice sets, each presenting 2 hypothetical surgical scenarios that varied in postoperative length of stay, patient’s preference, specialists’ experience, costs, national guideline recommendations, and carbon footprint. The questionnaire explored attitudes, behaviors, and barriers to environmental sustainability.

**Results:**

Among the 116 respondents, patient’s preference emerged as the most important factor in the choice of a surgical technique [relative importance (RI) 27.35 (95% CI: 19.57–35.12], followed by postoperative length of stay [RI 21.41 (95% CI: 14.07–28.75)], specialists’ experience [RI 16.07 (95% CI: 11.45–20.69)], costs [RI 13.98 (95% CI: 8.42–19.55)], national guideline recommendations [RI 13.29 (95% CI: 8.41–18.16)] and carbon footprint [RI 7.90 (95% CI: 3.63–12.18)]. Respondents expressed concern about climate change (105/116; 90%), with 85% (98/116) altering personal behaviors, and 49% (57/116) changing work practices. They feel surgical specialists have a responsibility to be aware of surgery’s environmental impact (97/116; 84%), and a part has the knowledge to decrease this impact (39/116; 34%). The main barriers are time (73/116; 63%), costs (74/116; 64%), and inadequate training and information (67/116; 58%).

**Conclusions:**

Patient preference and postoperative length of stay are prioritized in surgical decision-making, while carbon footprint is the least significant factor. To enhance sustainability in surgical practice, barriers must be addressed, and sustainable techniques and devices should be developed and standardized, ensuring sustainability is inherent in the available options, rather than just relying on individual choice.

Climate change is widely recognized as one of the greatest threats to global health in the 21st century.^1^ The impacts of global warming are expected to significantly affect health outcomes for populations worldwide, underscoring the critical role of the health care sector in addressing these challenges. The health care sector is responsible for ~4% of the global carbon footprint.^2^ Notably, the operating room (OR) contributes substantially to this environmental burden, generating roughly 20% to 30% of hospital waste.^3^

The ongoing development of surgical technologies and materials, including the increased use of disposables, contributes to the rising carbon footprint and growing volumes of medical waste.^4^ At the same time, technological advancements provide clinicians with a broader range of options, such as different approaches to performing a hysterectomy.^5^ However, robotically assisted laparoscopy has been shown to have a significantly higher intraoperative carbon footprint compared with conventional laparoscopy and laparotomy, primarily due to greater energy consumption and extensive disposable usage.^6,7^ Although these studies provide health care professionals with valuable insights into the environmental impact of their choices, it remains unclear to what extent the carbon footprint is considered in the decision-making process for surgical techniques and what priority it holds in clinical practice.

In the decision-making process for surgeries, health care professionals consider factors such as guidelines, experience and costs.^8^ Currently, no data are available about factors influencing the choice of surgical technique by surgical specialists and the role of the carbon footprint in this decision-making process.

Our study aims to elicit the significance of various factors influencing surgical specialists’ choices of specific surgical techniques and to determine the extent to which the carbon footprint is considered in their decision-making. By exploring the trade-offs and considerations that specialists make, we aim to offer unique insights into their decision-making processes. Correspondingly, the study will investigate the attitudes, behaviors, and barriers associated with integrating sustainable practices within clinical surgical settings.

## METHODS

Our study was conducted in the Netherlands and Dutch-speaking regions of Belgium using an online survey developed with Sawtooth Software (Sawtooth Software. Orem, UT). Respondents were recruited between February 2023 and May 2024.

### Study Design

A cross-sectional study with a survey of surgeons, gynecologists and urologists was performed. Respondents were eligible if they had experience with minimally invasive surgery (MIS). The online survey consisted of asking respondent characteristics, a discrete choice experiment (DCE), and a questionnaire to assess respondents’ attitudes, behaviors, and perceived barriers related to environmental sustainability. The DCE was conducted to assess the importance of different factors, further referred to as attributes, when surgeons, gynecologists, and urologists choose a specific surgical technique (robot-assisted laparoscopic, conventional laparoscopic, or open surgery).

### Survey Development

Identification of influential factors in the decision-making process, the attributes, is a critical component in the design of a DCE. For our study, attributes and their levels were selected according to the conceptual framework outlined by Helter and Boehler,^9^ which provides a structured 4-stage process for identifying and deriving necessary attributes and levels.

To ensure the DCE remained manageable for respondents, a maximum of 7 attributes was targeted.^10^ A detailed explanation of the attribute selection process is provided in the Supplementary Material, Supplemental Digital Content 1, http://links.lww.com/SLA/F431.

The first step involved identifying attributes through a literature review as part of stage 1: raw data collection. The literature review focused on robot-assisted laparoscopic surgery, conventional laparoscopic surgery, and open surgery. This stage resulted in 76 potential attributes. Following this, we used these attributes in semistructured interviews with 10 medical specialists (4 surgeons, 4 gynecologists, and 2 urologists). Participants were asked to identify the factors that influence their choice of surgical technique, considering what is important from the perspectives of the surgeon, patient, and society. In addition, they were presented with a refined list of attributes (attributes were categorized, and duplicates and overlapping items were merged or removed) and invited to suggest additional factors for inclusion.

In stage 2: Data reduction, attributes from the literature review and semistructured interviews were refined and combined to 21 based on input from experts. Stage 3: Removing inappropriate attributes involved a ranking exercise developed using Qualtrics XM Support, completed by 49 of the 62 surgical specialists approached, yielding a response rate of 79%. This exercise was designed to rank the most important attributes and eliminate less relevant ones. Finally, in stage 4: Wording, the researchers combined the ranking results with their expert judgment to select the final attributes for the DCE (Table S1, Supplemental Digital Content 1, http://links.lww.com/SLA/F431). These 6 attributes were divided into levels, determined based on literature data of 3 surgical techniques (open, conventional laparoscopic, and robot-assisted laparoscopic), which were used to create various surgical scenarios for the DCE.

The DCE started with a brief explanation and the presentation of a reference case to guide respondents in completing the choice tasks (Table S1, Supplemental Digital Content 1, http://links.lww.com/SLA/F431). Subsequently, respondents were asked to evaluate 14 choice tasks, each presenting 2 hypothetical surgical scenarios with varying levels of the attributes identified. For each choice task, respondents were required to select their preferred scenario from the 2 options provided. An example choice task is shown in Supplementary Figure 1, Supplemental Digital Content 1, http://links.lww.com/SLA/F431.

The final part of the survey included a questionnaire designed to assess respondents’ attitudes, behaviors, and perceived barriers related to environmental sustainability. The questionnaire was originally developed in a previous study,^11^ but was translated and adapted for this context using established cross-cultural adaptation guidelines.^12^ The adapted questionnaire and its translation are provided in the supplementary materials, Supplemental Digital Content 1, http://links.lww.com/SLA/F431. The lay-out and formulations were pilot tested by 2 surgical specialists before it was distributed.

### Data Collection

Sample size calculation for DCEs is a developing field. Hall et al^13^ proposed that 20 to 30 respondents per choice set can yield precise parameter estimates, while Lancsar and Louviere^14^ noted that typically no more than 20 observations per choice set are needed to estimate a reliable model. Marshall et al^15^ suggested a sample size of 100 to 300 respondents is appropriate in health care settings, considering resource constraints and potential limitations in participation rates due to specific medical conditions. In our study, which includes 14 choice tasks, we targeted a sample of 100 to 300 specialists to ensure robust analysis and the ability to study subgroups.

Respondents were recruited through newsletters distributed by scientific committees and via direct email outreach. To comply with privacy regulations, direct email addresses were limited, and the majority of invitations were disseminated indirectly via professional societies. To minimize selection bias, the survey was presented as research on the implementation of new technologies and techniques within MIS, without explicitly mentioning the sustainability aspect. The primary objective of our study was described as identifying the factors influencing a surgical specialist’s decision when selecting a surgical technique.

### Statistical Analysis

Hierarchical Bayes estimation was employed to calculate the relative importance (RI) of each attribute for each respondent based on the choices made in the choice tasks.^16^ The RI values were then averaged across all respondents to determine which attributes are, on average, most significant for surgical specialists when selecting a surgical technique.

All DCE data were analyzed using the Sawtooth Software Lighthouse Studio 9, version 9.15.0. (2024 Sawtooth Software Inc). Data regarding the environmental sustainability questions were exported from Sawtooth and analyzed in Microsoft Excel (Version 2308 Build 16.0.16731.20542).

### Ethics

The study has been approved for exemption from review by the Medical Ethics Review Committee (reference number N21.156). Informed consent was obtained at the beginning of the survey.

## RESULTS

In total, 116 respondents completed the survey. Table 1 shows the characteristics of the respondents.

**TABLE 1:** Baseline Characteristics of Respondents

Characteristic	Category	n=116	%
Gender	Male	62	53.4
	Female	54	46.6
	Other	0	0.0
Age (y)	Mean	46.08	
	SD	9.24	
Area of specialization	Urologist	29	25.0
	Surgeon	33	28.5
	Gynecologist	54	46.6
Country	The Netherlands	93	80.2
	Belgium	23	19.8
No. performed robotic-assisted laparoscopies (<1 y)	0–25	86	74.1
	25–50	13	11.2
	50–75	5	4.3
	75–100	5	4.3
	>100	7	6.0
No. performed laparoscopies (<1 y)	0–25	27	23.3
	25–50	29	25.0
	50–75	27	23.3
	75–100	12	10.3
	>100	21	18.1
No. performed laparotomies (<1 y)	0–25	78	67.2
	25–50	18	15.5
	50–75	12	10.3
	75–100	3	2.6
	>100	5	4.3

Regarding the choices made in the DCE, the patient’s preference [relative importance (RI) 27.35 (95% CI: 19.57–35.12)] is most important, followed by postoperative length of stay [RI 21.41 (95% CI: 14.07–28.75)], and the surgical specialist’s experience with the surgical technique [RI 16.07 (95% CI: 11.45–20.69)] in the choice of a surgical technique (Table 2). The average utilities show how much an attribute level is preferred over the alternative, with higher numbers indicating a higher preference for that level. The greater the difference in utility between the most and least preferred levels, the higher the RI for that attribute. The cost of the surgery being 2000 euros (38.67) is, for example, more strongly preferred over the costs being 8000 euros (−44.46; difference 83.13), than the preference for the emissions of 20 kg CO_2_ (19.74) over 50 kg CO_2_ (−20.30; difference 40.04), resulting in a high RI of cost than of CO_2_ footprint. Due to small subgroup sample sizes (surgeons, gynecologists, urologists), comparisons between groups were not feasible.

**TABLE 2:** Selected Attributes, Constructed Levels and Overall Utilities With SD and Overall Relative Importance (RI) With 95% CI

Attributes	Levels	Average overall utility per level (SD)	Overall RI (95% CI)
Patient’s preference	Objection	−97.444 (25.805)	27.35 (19.57–35.12)
	Neutral	32.900 (11.532)	
	Preference	64.544 (24.490)	
Postoperative length of stay	1 d	61.329 (25.784)	21.41 (14.07–28.75)
	3 d	4.6794 (13.643)	
	5 d	−66.009 (21.700)	
Level of experience	I feel somewhat experienced	−55.449 (18.682)	16.07 (11.45–20.69)
	I feel experienced	16.695 (16.332)	
	I feel very experienced	38.754 (13.318)	
Costs	2000 Euro	37.672 (18.383)	13.98 (8.42–19.55)
	4000 Euro	16.345 (11.101)	
	6000 Euro	−9.557 (13.523)	
	8000 Euro	−44.460 (18.673)	
Recommendation according to the national guideline	Recommended type of surgical technique according to the guideline (gold standard)	38.691 (12.487)	13.29 (8.41–18.16)
	Alternative type of surgical technique according to the guideline (sufficient scientific evidence)	−2.091 (18.853)	
	Guideline does not recommend this type of surgical technique (no scientific evidence)	−36.600 (23.863)	
CO_2_ footprint	20 kg of CO_2_	19.736 (14.417)	7.90 (3.63–12.18)
	30 kg of CO_2_	7.106 (13.723)	
	40 kg of CO_2_	−6.547 (10.488)	
	50 kg of CO_2_	−20.295 (18.297)	

**FIGURE 1:**
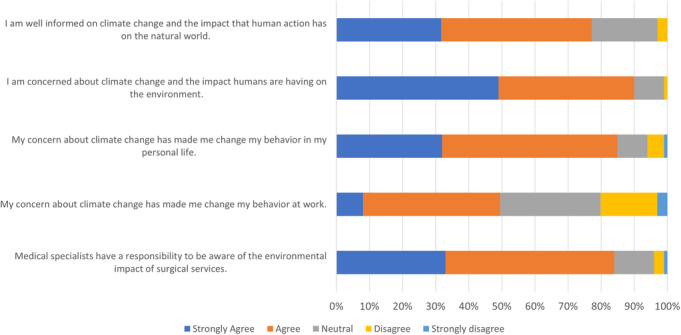
Attitudes regarding environmental sustainability (n=116).

In the questionnaire following the DCE, the respondents’ attitudes, behaviors, and perceived barriers related to environmental sustainability were assessed. All data are provided in the Supplementary Material, Supplemental Digital Content 1, http://links.lww.com/SLA/F431. Most respondents (105/116; 90%) agreed they were concerned about the threat of climate change and believed specialists should be aware of the environmental impact of surgery (97/116; 84%) (Fig. 1).

Most respondents changed their personal behavior due to climate change concerns (98/116; 85%) (Table S2, Supplemental Digital Content 1, http://links.lww.com/SLA/F431), primarily by recycling (102/116; 88%), reducing disposables and plastics (90/116; 78%), consuming less meat and dairy (83/116; 72%), and using public transport, biking, or walking to travel (73/116; 63%). In addition, respondents reported flying less or compensating for flights (69/116; 59%), owning an electric or hybrid car (41/116; 35%), volunteering or donating to a sustainable group (23/116; 20%), or being a member of an environmental or sustainability group (18/116; 16%).

**FIGURE 2:**
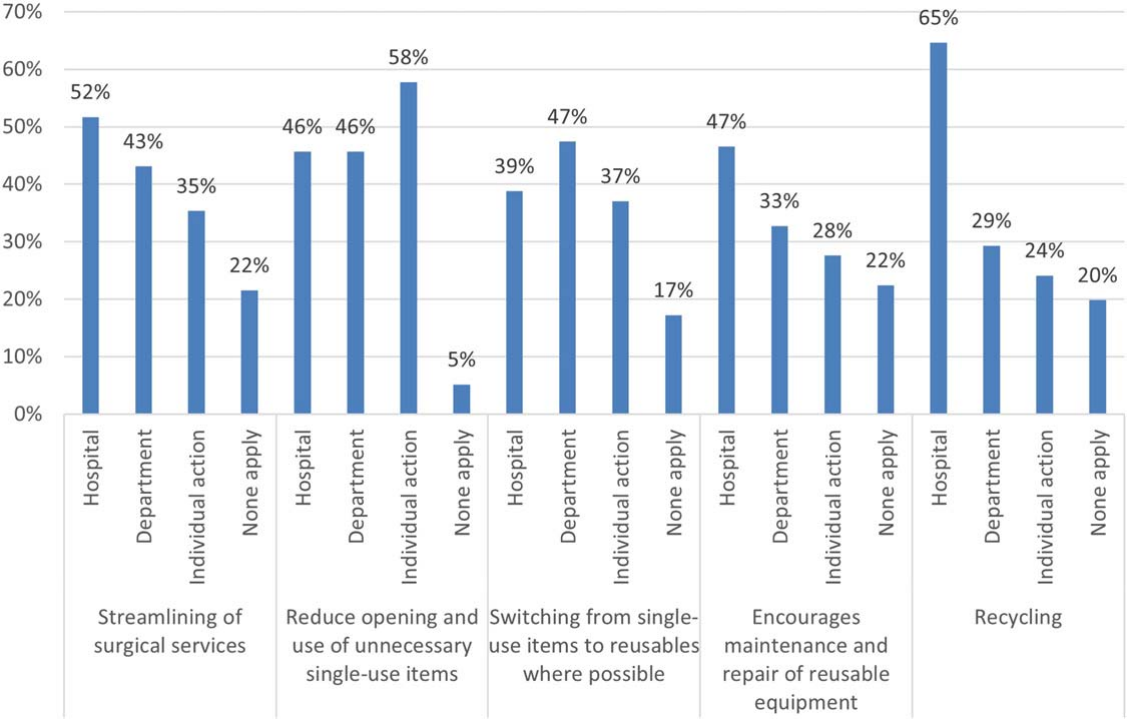
Individual behavior in encouraging sustainable actions: No action versus actions at individual, departmental, or hospital level (n=116).

At work, half of the respondents changed their behavior (57/116; 49%) (Table S2, Supplemental Digital Content 1, http://links.lww.com/SLA/F431), mainly by reducing the use of disposables (67/116; 58%), opting for reusable materials (43/116; 37%), and streamlining surgical services (41/116; 35%) (Fig. 2, Table S3, Supplemental Digital Content 1, http://links.lww.com/SLA/F431). Respondents were also asked whether actions were taken at the departmental or hospital level, or if no action was taken. On a hospital level recycling (75/116; 65%) and streamlining of surgical services (60/116; 52%) were greatly encouraged, and at the departmental level efforts such as switching to reusables (55/116; 47%), reducing opening and use of unnecessary single-use items (53/116; 46%), and streamlining of surgical services (50/116; 43%) were encouraged (Fig. 2, Table S3, Supplemental Digital Content 1, http://links.lww.com/SLA/F431). Most respondents felt supported to implement sustainability changes by their department (79/116; 68%), colleagues (83/116; 72%) and hospital (72/116; 62%) (Table S4, Supplemental Digital Content 1, http://links.lww.com/SLA/F431).

Thirty-four percent of respondents stated having enough knowledge to make surgery more sustainable (39/116; 34%), while 37% disagreed, and 29% remained neutral (Table S4, Supplemental Digital Content 1, http://links.lww.com/SLA/F431). More support from national authorities is preferred (80/116; 69%). Furthermore, a majority wants more oversight and regulation from local and/or national authorities to make the surgical practice more sustainable (74/116; 64%); however, a proportion of the respondents also disagree or strongly disagree (23/116; 20%) (Table S4, Supplemental Digital Content 1, http://links.lww.com/SLA/F431).

Respondents were asked about the availability and quality of education and training on sustainability (Table S3, Supplemental Digital Content 1, http://links.lww.com/SLA/F431). Some had received education at conferences (37/116; 32%), clinical review sessions (32/116; 28%), continuing education, courses, and (online) training organized by the hospital or department (18/116; 16%), outside the hospital or department (24/116; 21%), or as part of the medical curriculum (6/116; 5%). Among the 37 respondents who attended sustainability education at conferences, the majority (35; 95%) found them helpful (Table S3, Supplemental Digital Content 1, http://links.lww.com/SLA/F431). Notably, of those who had not attended, 39 respondents (49%) were interested if made available, whereas 10 respondents (13%) were not interested, and 25 respondents (32%) were unsure about their interest. See for more information on the other ways of education Table S3, Supplemental Digital Content 1, http://links.lww.com/SLA/F431.

**FIGURE 3:**
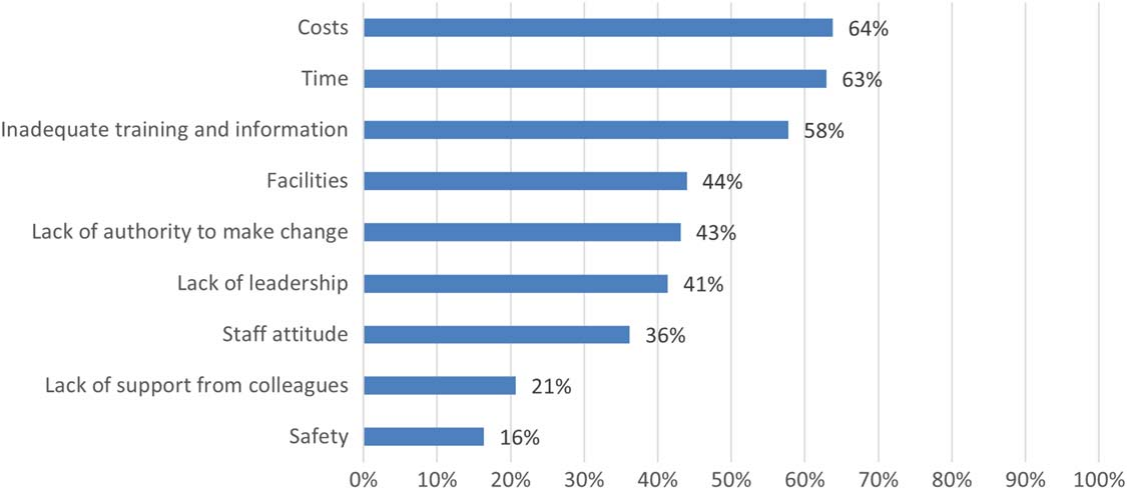
Potential barriers to sustainability efforts (n=116).

The main barriers to integrating sustainability are costs, time, and inadequate training and information (Fig. 3). Other reported challenges involve facility limitations, lack of authority, insufficient leadership support, and staff attitude. A minority of respondents noted concerns about lack of support from colleagues and safety.

To make surgery more environmentally sustainable, most respondents are willing to change personal practices (102/116; 88%), engage in relevant training and education (92/116; 79%), and join a (surgical) Green Team (64/116; 55%) (Table S4, Supplemental Digital Content 1, http://links.lww.com/SLA/F431). Respondents indicated that they would also want to target a proportion of their research, audit, or quality improvement efforts toward sustainability (43/116; 37%), become a sustainability frontrunner in their field (34/116; 29%), use educational budget towards relevant education (31/116; 27%), and only a small part would take no action (3/116; 3%).

## DISCUSSION

Our study is the first to explore the factors influencing the choice of a surgical technique and the role of the carbon footprint in this decision-making process. Patient preference is considered paramount, while the carbon footprint receives the lowest priority. Surgical specialists are willing to accept a higher carbon footprint in exchange for benefits such as lower costs or shorter postoperative recovery times. These findings highlight the risk-benefit trade-offs made in surgical decision-making, where carbon footprint is a less dominant factor. On the other hand, the respondents express concerns about climate change and an acknowledged responsibility to consider the environmental impact. Many surgical specialists are willing to make changes in their personal practices, and a substantial proportion are already taking action in surgical practice. However, inadequate training and information, lack of time, and expected costs appear to be barriers.

Considering patient preference, patients largely rely on doctors' advice, prioritizing effectiveness and side effects or complications.^17^ This underscores the role of surgical specialists in incorporating the carbon footprint into the decision-making process in cases where patient outcomes are comparable across surgical techniques. The results of our study reveal that surgeons prioritize patient preference over long-term consequences, such as the carbon footprint. These long-term effects are crucial not only to the individual patient’s health but also to broader public health outcomes and future generations. However, if patients were to prioritize a lower carbon footprint over a particular technique, it could provide an additional compelling factor in the decision-making process. These various factors must be carefully balanced to ensure patient safety is not compromised while also considering the broader implications for societal health.

Our study shows the carbon footprint often has a lower priority in selecting surgical techniques, raising the question of how to make surgery more sustainable when carbon footprint considerations are not or cannot be prioritized. Designing surgical techniques and instruments with sustainability in mind can shift responsibility away from specialists. Some specialists are motivated to consider sustainability but may lack the necessary guidance. Lack of knowledge is an underlying factor when time, costs, and insufficient training are mentioned as barriers. Sustainability initiatives do not necessarily increase costs; surgical specialists may be unaware of potential savings. In addition, sustainability efforts can be easily integrated if specialists have the knowledge of what is sustainable and how to implement this. Increasing education on sustainable practices could improve this, and some will still prioritize other factors—underscoring the value of incorporating sustainability into the design process itself.

Another identified key factor is the length of postoperative stay. A shorter postoperative stay not only benefits the patient but also contributes to a reduced carbon footprint.^18^ However, studies on the environmental impact of surgical techniques often focus only on the intraoperative period, neglecting postoperative outcomes and complications.^6,7^ Nevertheless, these studies on the environmental impact of surgical techniques provide valuable insights into improving the intraoperative sustainability of surgical techniques, such as reducing energy consumption, minimizing disposable materials, and extending the lifespan of surgical instruments.

Guidelines are crucial in promoting sustainability by incorporating environmental impact alongside clinical effectiveness. For instance, when evaluating surgical options for patients undergoing elective surgery for symptomatic inguinal hernia, it is important to assess how robot-assisted laparoscopic surgery compares to conventional laparoscopic and open surgery regarding carbon footprint. This ensures that environmental factors can be appropriately weighed alongside patient outcomes.

The costs of surgical techniques influence specialists’ choices more than the carbon footprint. However, there is often a lack of clarity regarding the specific costs of individual surgeries, and there are variations in costs between hospitals for the same procedures.^19,20^ This highlights the need for greater transparency and awareness regarding the financial implications of surgical procedures to support more informed decision-making in clinical practice.

The questionnaire used in our study was adapted from Harris et al,^11^ which surveyed Irish and UK surgeons. The proportions of respondents already implementing changes in their personal lives and workplaces were comparable across studies. However, a higher proportion of participants in our study reported having received education or training, potentially reflecting increased awareness in recent years. While Harris et al^11^ identified a lack of leadership and authority as key barriers, our respondents highlighted costs, time constraints, and inadequate training and information. Interestingly, a greater proportion of their respondents reported having adequate knowledge (51% vs 34% in our study). Addressing these barriers requires institutional leadership, with hospital boards fostering initiatives, providing resources, and approving costs where necessary.

A limitation of our study was the restriction on the number of attributes. Although we acknowledge surgical specialists consider numerous factors when selecting a surgical technique, we had to limit the attributes to conduct the DCE effectively. Therefore, we identified and included only the most influential factors. However, the hypothetical nature of the DCE scenarios may not fully capture the complexity of real-life decision-making. Factors such as institutional constraints, resource availability, and patient-specific clinical details likely also play a significant role, which were not accounted for in this study. This raises concerns about external validity, and future studies could complement our findings with observational or qualitative research. Another limitation was the sample size; while we aimed for 100 to 300 respondents, only 116 respondents completed all questions. This limited our ability to perform stratification and subgroup analyses. Variations in decision-making preferences across and within specialties, such as cancer versus non-cancer procedures or adult versus pediatric surgery, could not be explored. This issue may be attributed to the high dropout rate: 22 respondents were excluded for lacking MIS experience, 8 dropped out during the DCE explanation, and 43 respondents stopped during the choice tasks, potentially due to the number of choice tasks presented. To preserve the study’s relevance as a snapshot in time, we concluded data collection after slightly over a year. Extending the study further might have introduced bias, as increasing awareness of sustainability could have influenced the results. Finally, as the survey was disseminated anonymously, we were unable to conduct a nonresponder analysis. While this limits the ability to fully assess sample representativeness, our approach ensured compliance with data protection laws and allowed us to reach a wide range of surgeons.

The primary aim of our study was to elicit the preferences of surgeons, gynecologists, and urologists in their decision-making processes regarding surgical techniques, as well as to determine the significance of the carbon footprint in their choices. Despite climate change concerns, carbon footprint is the least significant factor in surgical decision-making. Addressing barriers could help align clinical practice with sustainability goals.

To facilitate this alignment, it is essential to develop inherently sustainable surgical techniques and devices, which would eliminate the need for clinicians to specifically choose eco-friendly options and, in turn, improve the environmental impact of surgical practices. Sustainability should also be a continuous consideration in health care, with practices such as replacing disposables with reusables, selecting low-impact techniques, and supporting clinical guidelines that advocate sustainable decision-making. It is particularly important to emphasize the incorporation of sustainable recommendations into clinical guidelines across various countries, especially where medical outcomes are comparable. This could provide a unified approach to promoting sustainability in health care and ensure that sustainability is considered alongside patient outcomes. Moreover, allocating time for health care professionals to focus on sustainability initiatives is crucial for reducing the carbon footprint in surgery and fostering a culture of sustainability within health care institutions. Institutional leadership plays a pivotal role in this process, as hospital boards and managers are responsible for supporting sustainability initiatives, providing necessary resources, and approving costs where needed. Finally, increasing education on environmental sustainability among surgical specialists is essential for creating meaningful change. Environmental sustainability should be incorporated as a standard component of the medical curriculum, the training of specialists, and the ongoing education of current surgical specialists. Continuous education ensures that health care providers are equipped with the knowledge and skills needed to effectively integrate sustainability into their practice.

In conclusion, while patient preferences and postoperative length of stay are the primary factors guiding surgical decision-making, the carbon footprint remains the least prioritized consideration. To promote greater sustainability in surgical practice, benefiting both societal health and future generations, it is essential to address existing barriers and concentrate on the development and standardization of sustainable techniques and devices. By ensuring that sustainability is inherently integrated into the available options, rather than relying solely on individual choice, we can foster more environmentally responsible decision-making in surgery.

## Supplementary Material
