# Supplementary material for: Decision-making Factors in Surgical Techniques and Attitudes Towards Environmental Sustainability
Source: Ann Surg. 2025 Mar 11;284(2):281–7. doi: 10.1097/SLA.0000000000006691 (PMC13344403; doi:10.1097/SLA.0000000000006691)
Supplement: Supplementary file 1 [file sla-284-281-s001.docx]

# Supplementary material

## DCE Data

### Detailed explanation of the attribute selection process

Attributes and their levels were selected according to the conceptual framework outlined by Helter and Boehler^9^, which provides a structured four-stage process for identifying and deriving necessary attributes and levels. To ensure the DCE remained manageable for respondents, a maximum of seven attributes was targeted.^10^

**Stage 1: Raw Data Collection.**The first step involved identifying potentially relevant attributes through a literature review as part of Stage 1. The literature review sought to find evidence on the characteristics of robot-assisted laparoscopies, conventional laparoscopies and laparotomies that might influence the choice of a surgical technique. A comprehensive literature search of Pubmed was undertaken in October 2021. The search included systematic reviews, meta-analyses and trials that were written in English, starting from the year 2000. Articles were selected if they included the comparison between robot-assisted laparoscopies, conventional laparoscopies and laparotomies.

In total 509 results were found. To limit the number of articles, all systematic reviews which compared robot-assisted laparoscopies, conventional laparoscopies and laparotomies from 2020 and 2021 were selected.

**Search strategy:***(("Robotic surgical procedures"[majr] OR "robotics"[ti] OR "robot assisted surgery"[ti] OR "robot surgery"[ti] OR "robot"[ti] OR "robot*"[ti]) AND ("Laparoscopy"[majr] OR "Laparoscopy"[ti] OR "Laparoscop*"[ti] OR "Laparotomy"[majr] OR "laparotomy"[ti] OR "laparotom*"[ti] OR "Hysterectomy"[majr] OR "hysterectomy"[ti] OR "hysterectom*"[ti] OR "Trachelectomy"[ti] OR "Trachelectom*"[ti] OR "minimally invasive"[ti] OR "Minimally Invasive Surgical Procedures"[majr:noexp]) AND ("Laparotomy"[majr] OR "Laparotomy"[ti] OR "Laparotom*"[ti] OR "open surgery"[ti] OR "open surg*"[ti] OR "open"[ti]) AND ("Comparative Study"[Publication Type] OR "Comparative Study" [tw] OR compar*[ti] OR systematic[sb] OR "meta-analysis"[pt] OR "meta analysis"[ti] OR "Clinical trial"[pt] OR "random*"[tw] OR "trial"[tw] OR "RCT"[tw]) AND english[la] AND ("2000/01/01"[PDAT] : "3000/12/31"[PDAT]))*

A total of 12 systematic reviews were included. The articles were reviewed, and potential attributes identified. We then refined these by categorising them, correcting for specific attributes, and removing duplicates.

| **Included articles** |
| --- |
| 1. Partelli S, Ricci C, Cinelli L, Montorsi RM, Ingaldi C, Andreasi V, Crippa S, Alberici L, Casadei R, Falconi M. Evaluation of cost-effectiveness among open, laparoscopic and robotic distal pancreatectomy: A systematic review and meta-analysis. Am J Surg. 2021 Sep;222(3):513-520. doi: 10.1016/j.amjsurg.2021.03.066. Epub 2021 Apr 7. PMID: 33853724. |
| 1. Aiolfi A, Lombardo F, Matsushima K, Sozzi A, Cavalli M, Panizzo V, Bonitta G, Bona D. Systematic review and updated network meta-analysis of randomized controlled trials comparing open, laparoscopic-assisted, and robotic distal gastrectomy for early and locally advanced gastric cancer. Surgery. 2021 Sep;170(3):942-951. doi: 10.1016/j.surg.2021.04.014. Epub 2021 May 20. PMID: 34023140. |
| 1. Park LS, Pan F, Steffens D, Young J, Hong J. Are Surgeons Working Smarter or Harder? A Systematic Review Comparing the Physical and Mental Demands of Robotic and Laparoscopic or Open Surgery. World J Surg. 2021 Jul;45(7):2066-2080. doi: 10.1007/s00268-021-06055-x. Epub 2021 Mar 26. PMID: 33772324. |
| 1. Ziogas IA, Evangeliou AP, Mylonas KS, Athanasiadis DI, Cherouveim P, Geller DA, Schulick RD, Alexopoulos SP, Tsoulfas G. Economic analysis of open versus laparoscopic versus robotic hepatectomy: a systematic review and meta-analysis. Eur J Health Econ. 2021 Jun;22(4):585-604. doi: 10.1007/s10198-021-01277-1. Epub 2021 Mar 19. PMID: 33740153. |
| 1. Aiolfi A, Lombardo F, Bonitta G, Danelli P, Bona D. Systematic review and updated network meta-analysis comparing open, laparoscopic, and robotic pancreaticoduodenectomy. Updates Surg. 2021 Jun;73(3):909-922. doi: 10.1007/s13304-020-00916-1. Epub 2020 Dec 14. PMID: 33315230; PMCID: PMC8184540. |
| 1. Raffone A, Travaglino A, Raimondo D, Boccia D, Vetrella M, Verrazzo P, Granata M, Casadio P, Insabato L, Mollo A, Seracchioli R. Laparotomic versus robotic surgery in elderly patients with endometrial cancer: A systematic review and meta-analysis. Int J Gynaecol Obstet. 2022 Apr;157(1):1-10. doi: 10.1002/ijgo.13766. Epub 2021 Jun 19. PMID: 34043235; PMCID: PMC9292514. |
| 1. Muaddi H, Hafid ME, Choi WJ, Lillie E, de Mestral C, Nathens A, Stukel TA, Karanicolas PJ. Clinical Outcomes of Robotic Surgery Compared to Conventional Surgical Approaches (Laparoscopic or Open): A Systematic Overview of Reviews. Ann Surg. 2021 Mar 1;273(3):467-473. doi: 10.1097/SLA.0000000000003915. PMID: 32398482. |
| 1. Ryan OK, Ryan ÉJ, Creavin B, Rausa E, Kelly ME, Petrelli F, Bonitta G, Kennelly R, Hanly A, Martin ST, Winter DC. Surgical approach for rectal cancer: A network meta-analysis comparing open, laparoscopic, robotic and transanal TME approaches. Eur J Surg Oncol. 2021 Feb;47(2):285-295. doi: 10.1016/j.ejso.2020.06.037. Epub 2020 Jul 26. PMID: 33280950. |
| 1. Tang W, Qiu JG, Deng X, Liu SS, Cheng L, Liu JR, Du CY. Minimally invasive versus open radical resection surgery for hilar cholangiocarcinoma: Comparable outcomes associated with advantages of minimal invasiveness. PLoS One. 2021 Mar 11;16(3):e0248534. doi: 10.1371/journal.pone.0248534. PMID: 33705481; PMCID: PMC7951922. |
| 1. Gavriilidis P, Roberts KJ, Aldrighetti L, Sutcliffe RP. A comparison between robotic, laparoscopic and open hepatectomy: A systematic review and network meta-analysis. Eur J Surg Oncol. 2020 Jul;46(7):1214-1224. doi: 10.1016/j.ejso.2020.03.227. Epub 2020 Apr 12. PMID: 32312592. |
| 1. Feng D, Li A, Hu X, Lin T, Tang Y, Han P. Comparative effectiveness of open, laparoscopic and robot-assisted radical cystectomy for bladder cancer: a systematic review and network meta-analysis. Minerva Urol Nefrol. 2020 Jun;72(3):251-264. doi: 10.23736/S0393-2249.20.03680-2. Epub 2020 Feb 19. PMID: 32083418. |
| 1. Balla A, Alarcón I, Morales-Conde S. Minimally invasive component separation technique for large ventral hernia: which is the best choice? A systematic literature review. Surg Endosc. 2020 Jan;34(1):14-30. doi: 10.1007/s00464-019-07156-4. Epub 2019 Oct 4. PMID: 31586250. |

From these studies, we identified potential attributes and categorised them.

| **Category** | **Attributes** |
| --- | --- |
| Costs | Cost-effectiveness, total costs, surgical procedure costs/operative costs, hospital stay costs, QALY |
| Pathological | Positive surgical margin, lymph node yields, resection rate, incomplete excision, circumferential resection margin (CRM), distal resection margin (DRM), tumour-free resection margin (R0) |
| Survival | Overall survival (OS), disease-free survival (DFS) |
| Oncological | Biochemical recurrence, disease recurring on image, local recurrence, regional recurrence, distant metastasis, overall recurrence |
| Peri-operative | Operative time, estimated blood loss (EBL), blood transfusion rate, minor complications, major complications, conversion to open procedure, insertion of indwelling catheter, nerve sparing procedure, intra-operative morbidity |
| Post-operative | General: re-operation, re-admission, overall morbidity, 30-day morbidity, overall mortality, 30-day mortality, severe postoperative complications (Clavien Dindo ≥ 3), return to work, length of time away from work, length of stay (LOS), time to regular diet  Infections: Intra-abdominal infection rate, wound infection rate, respiratory infection, abdominal sepsis, surgical site infection (SSI), minor infections, major infections  Cardiovascular: Thrombo-embolic complications, VTE, cardiovascular morbidity, cardiovascular complications, postoperative haemoglobin levels, postoperative bleeding, blood transfusion  Pulmonary: Pulmonary complications  Abdomen/Gastro-intestinal: parastomal/incisional hernia, bowel obstruction/ileus, time to oral diet, time to defecation, time to flatus, time to return of bowel function  Urogenital: urinary retention, urological injury, urinary function  Pain: postoperative opiate use, early postoperative pain assessed with the visual analogue scale, postoperative pain at rest, pain while performing regular activities, physical quality of life, pain and enhanced recovery  Neurovascular: preservation of the neurovascular and autonomic nerves |
| Surgeon | Comfort surgeon/ergonomics, mental impact/demand, visualization, fatigue, surgeon physiological tremor, visibility, improved movement of freedom, easy learning curve, dexterity, ability of scale motions/increased motion allowed by the instruments with multiple degrees of freedom |

Ultimately, we used the categories and attributes in semi-structured interviews with medical specialists. These interviews were conducted to gain more information on attributes that we should include. The semi-structured interviews were conducted with 10 medical specialists (four surgeons, four gynaecologists, and two urologists). Participants were asked to identify the factors that influence their choice of surgical technique, considering what is important from the perspectives of the surgeon, patient, and society. Additionally, they were presented with a condensed list of attributes and invited to suggest any further factors that could be incorporated. See the interview guide below.

**Interview guide:**
*Introduction*
Explanation of research on the implementation of new techniques in minimally invasive surgery, comparing robot-assisted surgery, laparoscopy, and laparotomy.

*General Information:*

- What is your position/field of expertise?
- What types of surgeries do you primarily perform, and what surgical techniques do you typically use? (e.g., laparoscopic, open, robot-assisted)
- How many years of experience do you have?

*Questions Related to Attributes*

- What factors influence your choice of a surgical technique?
- Does the type of surgery affect your choice? What factors lead you to choose differently?
- What is important to you from the perspective of the surgeon, the patient, and/or society?
- Is there anything else you would add to the list? (share list of attributes)

*List of Attributes:*

- Costs (e.g., surgical procedure costs, hospital stay costs)
- Oncological (e.g., resection margin, lymph node yield, survival, disease recurrence)
- Peri-operative (e.g., operative time, estimated blood loss (EBL), conversion to open)
- Post-operative (e.g., re-admission, return to work, length of stay (LOS), surgical site infection (SSI), sepsis, blood transfusion, ileus, urinary retention, pain)
- Surgeon (e.g., ergonomics, visibility, fatigue)

*Closing Question:*

- Does the sustainability of a surgical technique play a role in your choice of technique? If so, which aspects of sustainability do you consider?

The interviews were transcribed vertabim and attributes were coded using Atlas.ti. Additional attributes were added to the list of attributes that were derived from the literature review.

| **Categories** | **New attributes derived from interviews** |
| --- | --- |
| Costs | - |
| Pathological | - |
| Survival | - |
| Oncological | Anatomy (tumour contact/oncology) |
| Peri-operative | Incision length, blood loss, patient characteristics (BMI patient), fluorescence use |
| Post-operative | Postoperative stay, postoperative pain, functional recovery, quality of life, cosmetics, hernia, patient outcomes |
| Surgeon | Ergonomics, habituation surgeon, skills surgeon, visibility surgeon/resident, learning curve, training resident, heart rate variability |
| Surgery | Availability operating technique, indication, dexterity, operating time, skills team, location surgery, imaging techniques, Joined innovation robotics necessary as hospital |
| Sustainability | Sustainability (carbon footprint, pollution, material usage, waste, reuse/recycling of disposables, assessing actual needs (refuse), type of anesthesia, shortened hospital stay or no admission needed, energy consumption) |
| Patient | Patient Preference and Characteristics (patient wishes, surgical indication, contraindications, medical history, willingness to accept blood transfusions, religious beliefs, e.g., Jehovah’s Witnesses – no blood products) |
| Guidelines | National guidelines |

**Stage 2: Data reduction**

In the second stage, we refined the list of attributes based on expert advice, narrowing it down to 21 key attributes for use in the next stage.

Final attributes of this stage: Surgical costs, risk of mortality, cosmetic outcome, risk of conversion, quality of life (QoL), sexual function, bladder function, amount of blood loss, risk of blood transfusion, risk of surgical site infection (SSI), risk of readmission, postoperative hospital stay, time to functional recovery, time to recovery of bowel function, ergonomics of the surgeon, mental impact on the surgeon, level of experience of the surgeon, learning curve, carbon footprint, patient’s preference, guidelines/national agreements.

**Stage 3: Removing inappropriate attributes**

In the third stage we removed inappropriate attributes. We used the alternative method of simple rank ordering, whereby a group of informants rank the attributes in descending order of importance. We developed a questionnaire using Qualtrics XM Support, completed by 49 of the 62 surgical specialists approached, yielding a response rate of 79%. This exercise was designed to rank the most important attributes and eliminate less relevant ones.

Respondents were first asked to identify the five attributes they considered most important, followed by a question asking them to rate the importance of these attributes on a scale from 0 to 10.

*Choose 5 characteristics that are most important to you in the selection of a surgical technique.*

1. Influence of the surgery on quality of life (n=33)
2. Time to recovery of daily activities (n=33)
3. Level of experience with a specific surgical technique (n=29)
4. Postoperative hospital stay (n=21)
5. Guidelines/national agreements (n=19)
6. Patient’s preference (n=16)
7. Risk of conversion (n=16)
8. Surgical costs (n=11)
9. Ergonomics of the surgeon (n=11)
10. CO_2_ footprint (n=8)
11. Amount of blood loss (n=8)
12. Risk of SSI (n=8)
13. Risk of mortality (n=7)
14. Cosmetic outcome (n=4)
15. Time to recovery of bowel function (n=4)
16. Risk of readmission (n=3)
17. Influence of surgery on sexual function (n=3)
18. Risk of blood transfusion (n=2)
19. Improving the learning curve (n=2)
20. Influence of surgery on bladder function (n=1)
21. Mental impact on the surgeon during surgery (n=1)

*Please indicate how important you find each of these on a scale from 0 to 10.*

1. Influence of the surgery on quality of life (total weighing: 324)
2. Time to recovery of daily activities (315)
3. Level of experience with a specific surgical technique (282)
4. Postoperative hospital stay (198)
5. Guidelines/national agreements (175)
6. Patient’s preference (143)
7. Risk of conversion (140)
8. Ergonomics surgeon (97)
9. Surgical costs (87)
10. Risk of SSI (70)
11. Amount of blood loss (70)
12. Risk of mortality (70)
13. CO_2_-footprint (69)
14. Time to recovery of bowel function (36)
15. Cosmetic outcome (31)
16. Influence of surgery on sexual function (28)
17. Risk of readmission (26)
18. Risk of blood transfusion (19)
19. Improving the learning curve (15)
20. Influence of surgery on bladder function (9)
21. Mental impact on the surgeon during surgery (8)

| # | Attributes | Minimum | Maximum | Mean | Std deviation | Variance | Number | **Total weighing** |
| --- | --- | --- | --- | --- | --- | --- | --- | --- |
| 1 | Surgical costs | 6 | 11 | 7.91 | 1.44 | 2.08 | 11 | 87.01 |
| 2 | Risk of mortality | 8 | 11 | 10 | 1.07 | 1.14 | 7 | 70 |
| 3 | Cosmetic outcome | 7 | 8 | 7.75 | 0.43 | 0.19 | 4 | 31 |
| 4 | Risk of conversion | 7 | 11 | 8.75 | 1.03 | 1.06 | 16 | 140 |
| 5 | Influence of the surgery on quality of life | 8 | 11 | 9.82 | 0.97 | 0.94 | 33 | 324.06 |
| 6 | Influence of the surgery on sexual function | 9 | 10 | 9.33 | 0.47 | 0.22 | 3 | 27.99 |
| 7 | Influence of surgery on bladder function | 9 | 9 | 9 | 0.00 | 0 | 1 | 9 |
| 8 | Amount of blood loss | 5 | 11 | 8.75 | 1.56 | 2.44 | 8 | 70 |
| 9 | Risk of blood transfusion | 9 | 10 | 9.5 | 0.50 | 0.25 | 2 | 19 |
| 10 | Risk of SSI | 7 | 10 | 8.75 | 0.83 | 0.69 | 8 | 70 |
| 11 | Postoperative hospital stay | 8 | 11 | 9.43 | 0.73 | 0.53 | 21 | 198.03 |
| 12 | Time to recovery of daily activities | 8 | 11 | 9.55 | 0.99 | 0.98 | 33 | 315.15 |
| 13 | Time to recovery of bowel function | 9 | 9 | 9 | 0.00 | 0 | 4 | 36 |
| 14 | Ergonomics surgeon | 6 | 11 | 8.82 | 1.53 | 2.33 | 11 | 97.02 |
| 15 | Mental impact on the surgeon during surgery | 8 | 8 | 8 | 0.00 | 0 | 1 | 8 |
| 16 | Level of experience with a specific surgical technique | 7 | 11 | 9.72 | 1.01 | 1.03 | 29 | 281.88 |
| 17 | Improving the learning curve | 7 | 8 | 7.5 | 0.50 | 0.25 | 2 | 15 |
| 18 | CO2-footprint | 7 | 10 | 8.63 | 0.86 | 0.73 | 8 | 69.04 |
| 19 | Patient’s preference | 7 | 11 | 8.94 | 1.14 | 1.31 | 16 | 143.04 |
| 20 | Risk of readmission | 8 | 9 | 8.67 | 0.47 | 0.22 | 3 | 26.01 |
| 21 | Guidelines/national agreements | 7 | 11 | 9.26 | 1.16 | 1.35 | 19 | 175.94 |

**Stage 4: Wording**

Finally, we combined the ranking results with our expert judgment to select the final six attributes for the DCE (Supplementary table 1). These six attributes were divided into levels, determined based on literature data of three surgical techniques (open, conventional laparoscopic, and robot-assisted laparoscopic), which were used to create various surgical scenarios for the DCE.

### Supplementary Figure 1. Example choice task


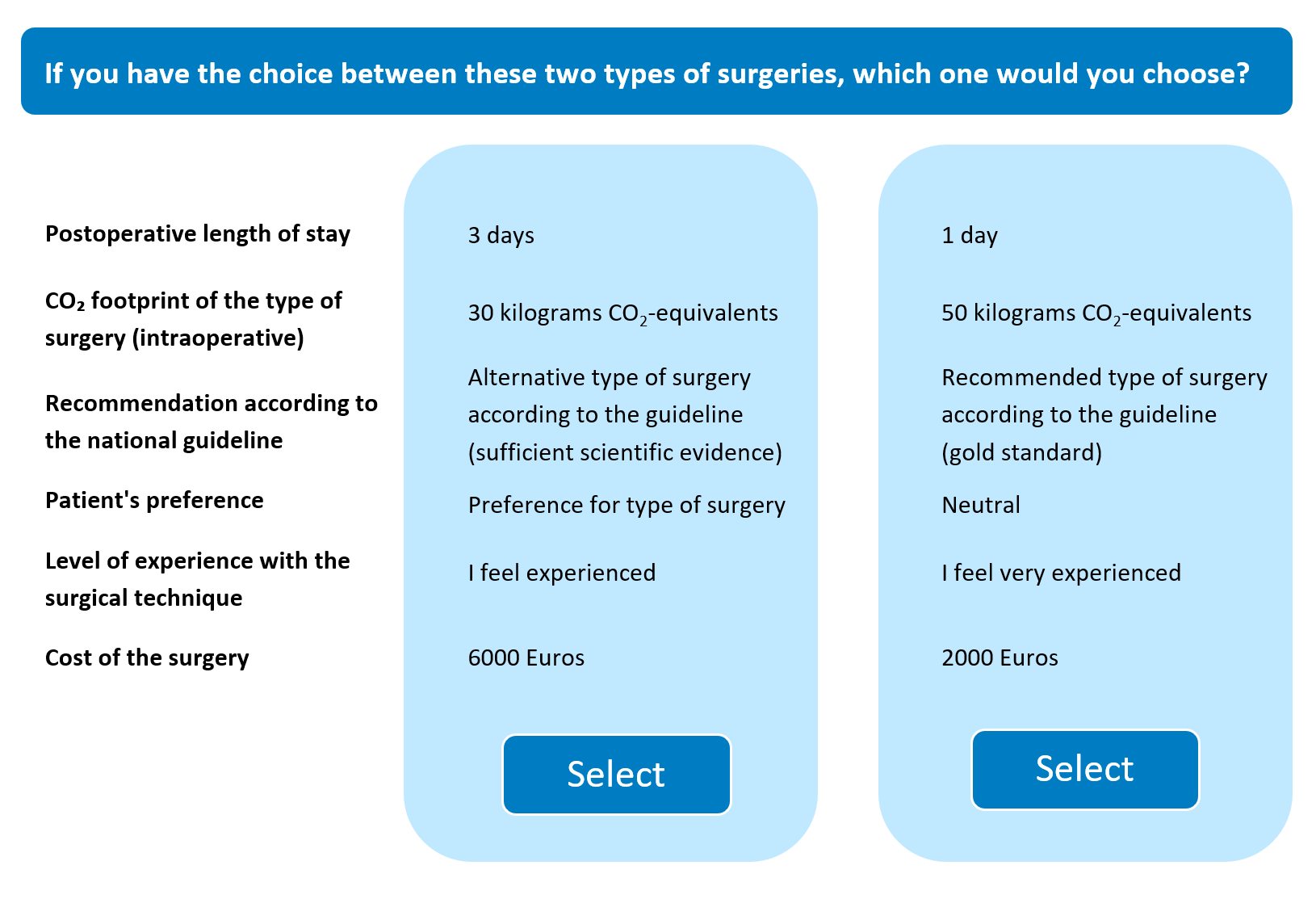


### Supplementary Table 1: Attributes, levels and reference case used in the discrete choice experiment

| **Attributes** | **Explanation** | **Levels** |
| --- | --- | --- |
| 1. Level of experience with the surgical technique | Surgeon's level of experience with the type of surgical technique | I feel somewhat experienced  I feel experienced  I feel very experienced |
| 1. Postoperative length of stay | The duration of the patient's hospital stay after the surgery | 1 day  3 days  5 days |
| 1. Recommendation according to the national guideline for this type of surgery | This is the national guideline, which outlines diagnostics, treatment, etc., based on scientific evidence for a particular diagnosis. | Recommended type of surgery according to the guideline (gold standard)  Alternative type of surgery according to the guideline (sufficient scientific evidence)  Guideline does not recommend this type of surgery (no scientific evidence) |
| 1. Patient's preference | This is the patient's preference regarding the type of surgery for their treatment. | Objection to type of surgery  Neutral  Preference for type of surgery |
| 1. Cost of the surgery | These are the costs of the surgery itself, including equipment (purchase and maintenance costs), disposable instruments, and operating room time costs. | 2000 Euro  4000 Euro  6000 Euro  8000 Euro |
| 1. CO₂ footprint of the type of surgery (from start to end of surgery) | This is the total emission of greenhouse gases from the type of surgery (from the beginning to the end of the surgery). | 20 kilograms of CO₂ *- equivalent to driving 70 kilometers with a gasoline car (from Amsterdam to Rotterdam)*  30 kilograms of CO₂ *- equivalent to driving 110 kilometers with a gasoline car (from Amsterdam to Tilburg)*  40 kilograms of CO₂ *- equivalent to driving 150 kilometers with a gasoline car (from Amsterdam to Antwerp)*  50 kilograms of CO₂ *- equivalent to driving 190 kilometers with a gasoline car (from Amsterdam to Brussels)* |
| **Reference case**  You are about to choose a type of surgery for one of your patients. The patient is a 45-year-old with no medical history, a normal BMI of 23, and no current medication use. The condition in question is benign. | | |

## Questionnaire data

### Supplementary Table 2: Current attitudes and values

| **Question** | **Response** | **Count** | **% (respondents for individual question)** |
| --- | --- | --- | --- |
| 1.A.1 I am well informed on climate change and the impact that human action has on the natural world | Strongly agree | 37 | 32% |
|  | Agree | 53 | 46% |
|  | Neutral | 23 | 20% |
|  | Disagree | 3 | 3% |
|  | Strongly disagree | 0 | 0% |
| 1.A.2 I am concerned about climate change and the impact humans are having on the environment | Strongly agree | 57 | 49% |
|  | Agree | 48 | 41% |
|  | Neutral | 10 | 9% |
|  | Disagree | 1 | 1% |
|  | Strongly disagree | 0 | 0% |
| 1.B If you disagreed or strongly disagreed with the statement: "I am concerned about climate change" please indicate your reasons below. (Please tick all that apply) | I am concerned that patient care could be compromised if climate change becomes a consideration | 0 | 0% |
|  | I don’t know enough about climate change for this to be a major concern | 0 | 0% |
|  | One person will not make a difference | 0 | 0% |
|  | These issues are not my responsibility | 0 | 0% |
|  | I have more important concerns | 0 | 0% |
|  | I don’t care about climate change | 1 | 1% |
|  | I do not believe climate change is real | 0 | 0% |
|  | The changes are too inconvenient | 0 | 0% |
| 2.A My concern about climate change has made me change my behaviour in my personal life | Strongly Agree | 37 | 32% |
|  | Agree | 61 | 53% |
|  | Neutral | 11 | 9% |
|  | Disagree | 6 | 5% |
|  | Strongly disagree | 1 | 1% |
| 2.B My behaviour has changed in the following way. (Please tick all that apply) | I recycle where possible at home | 102 | 88% |
|  | I try to minimise my use of single-use items and plastics at home | 90 | 78% |
|  | I try to consume low amounts of red meat and dairy produce | 83 | 72% |
|  | I use public and/or active transport to try to reduce my carbon footprint | 73 | 63% |
|  | I am trying to reduce my number of flights, or carbon offset these | 69 | 59% |
|  | I volunteer for or donate to an environmental charity | 23 | 20% |
|  | I am a member of an environmental group | 18 | 16% |
|  | I own an electric/hybrid car | 41 | 35% |
|  | I make no efforts | 0 | 0% |
| 3.1 My concern about climate change has made me change my behaviour at work | Strongly Agree | 9 | 8% |
|  | Agree | 48 | 41% |
|  | Neutral | 35 | 30% |
|  | Disagree | 20 | 17% |
|  | Strongly disagree | 4 | 3% |
| 3.2 Medical specialists have a responsibility to be aware of the environmental impact of surgical services | Strongly Agree | 38 | 33% |
|  | Agree | 59 | 51% |
|  | Neutral | 14 | 12% |
|  | Disagree | 4 | 3% |
|  | Strongly disagree | 1 | 1% |

### Supplementary Table 3: Current practice and education in the surgical workplace

| 4. Does your hospital or department encourage the following sustainability efforts, or do you seek to improve these through individual action? (Please tick all that apply)  Count (% respondents for individual question) | | | | | | |
| --- | --- | --- | --- | --- | --- | --- |
|  | Streamlining of surgical services | Reduce opening and use of unnecessary single-use items | Switching from single-use items to reusables where possible | Encourages maintenance and repair of reusable equipment | | Recycling |
| Hospital | 60  (52%) | 53  (46%) | 45  (39%) | 54  (47%) | | 75  (65%) |
| My department | 50  (43%) | 53  (46%) | 55  (47%) | 38  (33%) | | 34  (29%) |
| Individual action | 41  (35%) | 67  (58%) | 43  (37%) | 32  (28%) | | 28  (24%) |
| None apply | 25  (22%) | 6  (5%) | 20  (17%) | 36  (22%) | | 23  (20%) |
| 5. Please comment on the availability and quality of environmental education and training. (Please tick all that apply) Count (% respondents for individual question) | | | | | | |
|  | Conferences | Clinical review sessions | Continuing education, courses, and (online) training organised by the hospital or department | Continuing education, courses, and (online) training organised outside the hospital or department | Medical curriculum | |
| Attended and helpful | 35  (30%) | 29  (25%) | 15  (13%) | 22  (19%) | 6 (5%) | |
| Attended and not helpful | 2  (2%) | 3  (3%) | 3  (3%) | 2  (2%) | 0 (0%) | |
| Not available but would like to attend | 39  (34%) | 43  (37%) | 59  (51%) | 35  (30%) | 38 (33%) | |
| Not available and not interested | 5  (4%) | 7  (6%) | 4  (3%) | 4  (3%) | 3 (3%) | |
| Available but not interested in attending | 10  (9%) | 7  (6%) | 7  (6%) | 8  (7%) | 5 (4%) | |
| Do not know | 25  (22%) | 27  (23%) | 28  (24%) | 45  (39%) | 64 (55%) | |

### Supplementary Table 4: Future engagement with sustainable surgery, and perceived barriers to change

| 6. To what extent do you feel that the following would apply if you were trying to make a change directed towards sustainable surgery in your department?  Count (% respondents for individual question) | | | | |
| --- | --- | --- | --- | --- |
|  | I have the appropriate knowledge | I would have the support of my department | I would have the support of my colleagues | I would have the support of my hospital |
| Strongly agree | 8  (7%) | 17  (15%) | 16  (14%) | 20  (17%) |
| Agree | 31  (27%) | 62  (53%) | 67  (58%) | 52  (45%) |
| Neutral | 34  (29%) | 29  (25%) | 27  (23%) | 38  (33%) |
| Disagree | 39  (34%) | 7  (6%) | 6  (5%) | 4  (3%) |
| Strongly disagree | 4  (3%) | 1  (1%) | 0  (0%) | 2  (2%) |

| **Question** | **Response** | **Count** | **% (respondents for individual question)** |
| --- | --- | --- | --- |
| 7. In your opinion, what are the potential barriers to sustainability efforts? (Please tick all that apply) | Lack of leadership | 48 | 41% |
|  | Lack of authority to make change | 50 | 43% |
|  | Inadequate training and information | 67 | 58% |
|  | Staff attitude | 42 | 36% |
|  | Cost | 74 | 64% |
|  | Time | 73 | 63% |
|  | Facilities | 51 | 44% |
|  | Lack of support from colleagues | 24 | 21% |
|  | Safety | 19 | 16% |
| 8.1 To what extent do you agree with the following statement: ‘I would welcome more guidance from national bodies on how I can improve the sustainability of my own surgical practice’ | Strongly Agree | 27 | 23% |
|  | Agree | 53 | 46% |
|  | Neutral | 25 | 22% |
|  | Disagree | 7 | 6% |
|  | Strongly disagree | 4 | 3% |
| 8.2 To what extent do you agree with the following statement ‘I would welcome more monitoring and regulation from local and/ or national bodies to improve sustainability within surgical practice‘ | Strongly Agree | 23 | 20% |
|  | Agree | 51 | 44% |
|  | Neutral | 19 | 16% |
|  | Disagree | 14 | 12% |
|  | Strongly disagree | 9 | 8% |
| 9. To improve surgical sustainability I am willing to: | Engage in relevant training and education | 92 | 79% |
|  | Join a (surgical) Green Team | 64 | 55% |
|  | Use my educational budget towards relevant education | 91 | 27% |
|  | Target a proportion of my research, audit or quality improvement efforts towards sustainability | 43 | 37% |
|  | Become a green champion in my area | 34 | 29% |
|  | Make changes to my personal practice | 102 | 88% |
|  | No action | 3 | 3% |
